# Seroprevalence of Crimean-Congo hemorrhagic fever in humans in the World Health Organization European region: A systematic review

**DOI:** 10.1371/journal.pntd.0008094

**Published:** 2020-03-02

**Authors:** Lía Monsalve-Arteaga, Montserrat Alonso-Sardón, Juan Luis Muñoz Bellido, María Belén Vicente Santiago, María Carmen Vieira Lista, Julio López Abán, Antonio Muro, Moncef Belhassen-García

**Affiliations:** 1 Laboratorio de Inmunología Parasitaria y Molecular, Centro de Investigación de Enfermedades Tropicales de la Universidad de Salamanca (CIETUS), Instituto de Investigación Biomédica de Salamanca (IBSAL), Facultad de Farmacia, Universidad de Salamanca, Salamanca, Spain; 2 Área de Medicina Preventiva y Salud Pública, CIETUS, IBSAL, Facultad de Medicina, Universidad de Salamanca, Salamanca, Spain; 3 Servicio de Microbiología y Parasitología, Complejo Asistencial Universitario de Salamanca, Salamanca, Spain; 4 Departamento de Ciencias Biomédicas y del Diagnóstico, Universidad de Salamanca, Salamanca, Spain; 5 Instituto de Investigación Biomédica de Salamanca (IBSAL), Universidad de Salamanca, CSIC, Complejo Asistencial Universitario de Salamanca, Salamanca, Spain; 6 Servicio de Medicina Interna. Sección de Enfermedades Infecciosas. CAUSA. CIETUS. IBSAL. Universidad de Salamanca, Salamanca, Spain; University of Minnesota, UNITED STATES

## Abstract

**Background:**

Crimean-Congo hemorrhagic fever (CCHF) is an emerging infectious disease caused by a *Nairovirus*. CCHF is a tick-borne disease that is predominantly associated with *Hyalomma* ticks and have a widespread distribution in Africa, Asia and Europe. CCHF usually presents as a subclinical disease, but in some cases, it may present as a hemorrhagic fever with a high mortality rate. This systematic review of the literature was performed to identify the available evidence on the prevalence of CCHF in the European Region of the World Health Organization, based on seroprevalence (IgG antibodies).

**Methodology:**

A systematic review was performed following the Preferred Reporting Items for Systematic Reviews and Meta-Analyses (PRISMA) statement protocol. PubMed, Embase, and the Web of Science were used for the search (up to January 31, 2019), combining the following MeSH terms: [“Crimean-Congo haemorrhagic fever” OR “Crimean-Congo hemorrhagic fever virus” OR “Congo-Crimea” OR “Crimea-Congo”] AND [“Europe”] AND [“epidemiology” OR “seroprevalence”]. The abstracts were screened. Subsequently, full-text articles were selected and reviewed based on the PICOS (Population-Intervention-Comparison-Outcomes-Study type) criteria by two independent reviewers for inclusion in the final analysis. The data were qualitatively synthesized without quantitative pooling due to the heterogeneity in the study populations and methodologies.

**Principal findings:**

Thirty articles (9 from western Europe, 18 from central Europe and 3 from eastern Europe) were included in the analysis. All articles were cross-sectional studies (descriptive studies).

**Conclusions:**

The highest seroprevalence of CCHF is found in central and eastern European countries. Southern and western Europe countries, such as Greece and Spain, have low levels of endemicity, but the spread of the infection, which is associated with climate change, is a possibility that we should keep in mind. Further studies, especially larger seroprevalence studies in humans and animals, are needed to establish the current status of the CCHF epidemiology and to generate standardized guidelines for action in the region.

## Author summary

Crimean Congo hemorrhagic fever (CCHF) is a widespread tick-borne viral disease caused by a Nairovirus of the *Nairoviridae* family. CCHF-virus (CCHFV) has been considered to be one of the eight priority emergent pathogens for the last 3 years by the World Health Organization (WHO), requiring urgent attention in Research, Development and Innovation (R&D&I) because of its epidemic potential in the near future In this systematic review, we aimed to describe the epidemiological impact of CCHFV (seroprevalence for IgG antibodies and the associated risk factors) in the WHO European Region (WHO/Europe). In this systematic review suggests the following conclusions. i) The highest values of CCHFV seroprevalence are found in Turkey, the Russian Federation, and Kazakhstan. ii) Greece has a high seroprevalence, though only one death associated with CCHFV has been reported. This fact contrasts with the neighboring countries, such as Balkan countries and Turkey, where the rate of severe infections seems to be higher. iii) Extensive studies should be developed in European countries to establish the actual epidemiological situation and to take additional preventive measures for the future.

## Introduction

Crimean Congo hemorrhagic fever (CCHF) is a widespread tick-borne viral disease caused by CCHFV of the family Nairoviridae [1]. This disease was first described in 1944, during World War II, when an outbreak affected a group of Soviet soldiers in the Crimean Peninsula [2]. Twenty years later, in 1967, the virus was finally identified and was named Crimean-Congo virus, based on the similarities found with the virus that affected a febrile patient in the former Belgian Congo in 1956 [3].

Ninety percent of Crimean-Congo virus infections are oligosymptomatic or asymptomatic[4]. In the remaining 10%, the infections can present as a severe disease with a higher mortality rate [5–7]. Mortality is associated with different factors, such as age, viral strain, and endemicity [8–11].

The transmission of this virus to humans is mainly associated with the bite of hard-bodied ticks (*Ixodidae* family), predominantly those belonging to the genus *Hyalomma*, which are widely distributed in Asia, Africa and Europe. The infection can also be acquired through direct contact with blood and other bodily fluids from infected animals and humans, mainly those with a high viral load, including hospitalized patients with hemorrhagic fever. Thus, there is a high risk of transmission in healthcare environments [8,12–14]. Currently, the CCHF virus (CCHFV) is considered a level 4 biosecurity risk pathogen by the Centers for Disease Control and Prevention (CDC) [15,16]. CCHFV has been considered to be one of the eight priority emergent pathogens for the last 3 years by the World Health Organization (WHO), requiring urgent attention in Research, Development and Innovation (R&D&I) because of its epidemic potential in the near future [17,18].

Almost 1000 cases of CCHFV infection are reported in the Middle East and eastern European countries yearly [11,19]. In Europe, human cases have been reported in Albania, Bulgaria, Kosovo, Russia, Serbia, Turkey, Ukraine, Greece, Georgia and Spain [1,20,21].

The aim of this study was to identify the epidemiological impact of CCHFV (seroprevalence for IgG antibodies and the associated risk factors) in the WHO European Region, through a systematic review, to address the research question: what is the seroprevalence of CCHFV infection in the different geographic areas of Europe, and what are the possible associated risk factors?

## Material and methods

### Study design

This systematic review was conducted in accordance with the Preferred Reporting Items for Systematic Reviews and Meta-Analyses (PRISMA) statement [22]. Study eligibility was defined according to the conventional PICOS (Population-Intervention-Comparison-Outcomes-Study type) criteria [23,24], which were determined *a priori*, including the following: Population (CCHFV-seropositive individuals); Intervention or Exposure, (risk factors, environmental determinants facilitating and inhibiting viral transmission); Comparators (three geographical regions of Europe, western/central/eastern Europe); Outcomes (seroprevalence data, IgG antibodies); and Study design (Observational studies, descriptive and analytical designs).

### Search strategy and selection criteria

A systematic review of electronic bibliographic databases was performed for publications up to January 31, 2019. The following databases were searched for relevant studies to identify all the published studies about the seroprevalence of CCHFV in Europe: PubMed, Embase and the Web of Science, with the language restrictions of English, Spanish or French.

The electronic research was performed using the following Boolean operators and terms: ["Crimean Congo hemorrhagic fever" OR "Crimea Congo hemorrhagic fever" OR "Congo Crimea hemorrhagic fever" OR “Crimean Congo hemorrhagic fever virus” OR “Crimean Congo” OR “Crimea-Congo”] AND “Europe” AND [“seroprevalence” OR “epidemiology”].

**Inclusion criteria:** Published reports evaluating the epidemiology of CCHF in WHO/Europe were included when they fulfilled the following selection criteria, according to PRISMA guidelines. **(1)** Population: Human studies about the seroprevalence of CCHF. **(2)** Study design and interventions: We included observational studies. Randomized controlled trials were not included because these studies evaluate the efficacy of a treatment or an intervention. **(3)** Types of outcome measures: As the main outcome, we compared the seroprevalence of CCHF (defined as the number of individuals with evidence of IgG antibodies against CCHFV) in the 3 areas: western, central and eastern WHO/Europe.

**Exclusion criteria:** All nonhuman studies, intention-to-treat clinical trials, incidence data, editorial letters, letters to the editor, expert committees, author opinions and case reports (OCEBM Level of Evidence 5, Grade of Recommendation D) were excluded because they do not allow decision making or recommendation proposals and/or do not talk about an epidemiological observation. Finally, all studies about CCHF imported cases from an endemic to a nonendemic region were also excluded.

### Selection of studies, data collection/extraction, and data synthesis/analysis

For the critical evaluation of the quality of the included studies, we applied a uniform checklist method to identify the internal validity and possible bias. To identify and select the studies, we classified them in a table following a systematic method. We evaluated the quality of each study, and the conclusions were based on the evidence levels according to the Oxford Centre for Evidence-Based Medicine (OCEBM) [25], which allow us to judge the strength of evidence. Also, we used the recommendations of the PRISMA declaration as a guide.

We prepared tables to make the systematic collection of the qualitative and quantitative data of each article. All the data were compiled by the last name of the first author, the year of publication, country, study design, objective, individuals or patients studied, population characteristics and other risk factors and the seroprevalence of anti-CCHFV IgG antibodies. First, the articles were evaluated based on the title and abstract, and afterwards they were evaluated based on the full text. All studies that did not fulfill the inclusion criteria were excluded from this systematic review.

All the articles identified were revised by two review team members (LMA and MAS), which followed the methodological standards recommended by the Committee on Standards for Systematic Reviews of Comparative Effectiveness Research for finding and assessing individual study: worked independently, screened and selected studies and extracted quantitative and other critical data from included studies. Each eligible study was systematically appraised for risk of bias; relevance to the study’s populations, and outcomes measures: seroprevalence. All the discrepancies were resolved by the rest of the study team.

We initially included all the countries of the WHO/European Region, and then we separated these studies into three different subgroups corresponding to three European regions, based on demographic and epidemiologic factors, and we made a qualitative comparison between these three European regions. The 53 countries of the WHO European Region were subdivided into three geographical areas, based on epidemiological considerations and in accordance with the division used by World Health Organization (WHO) and European Centre for Disease Control (ECDC) in others reports on surveillance in Europe: West (23 countries), Centre (15 countries) and East (15 countries).

## Results

### Summary of the included articles

**Fig 1:**
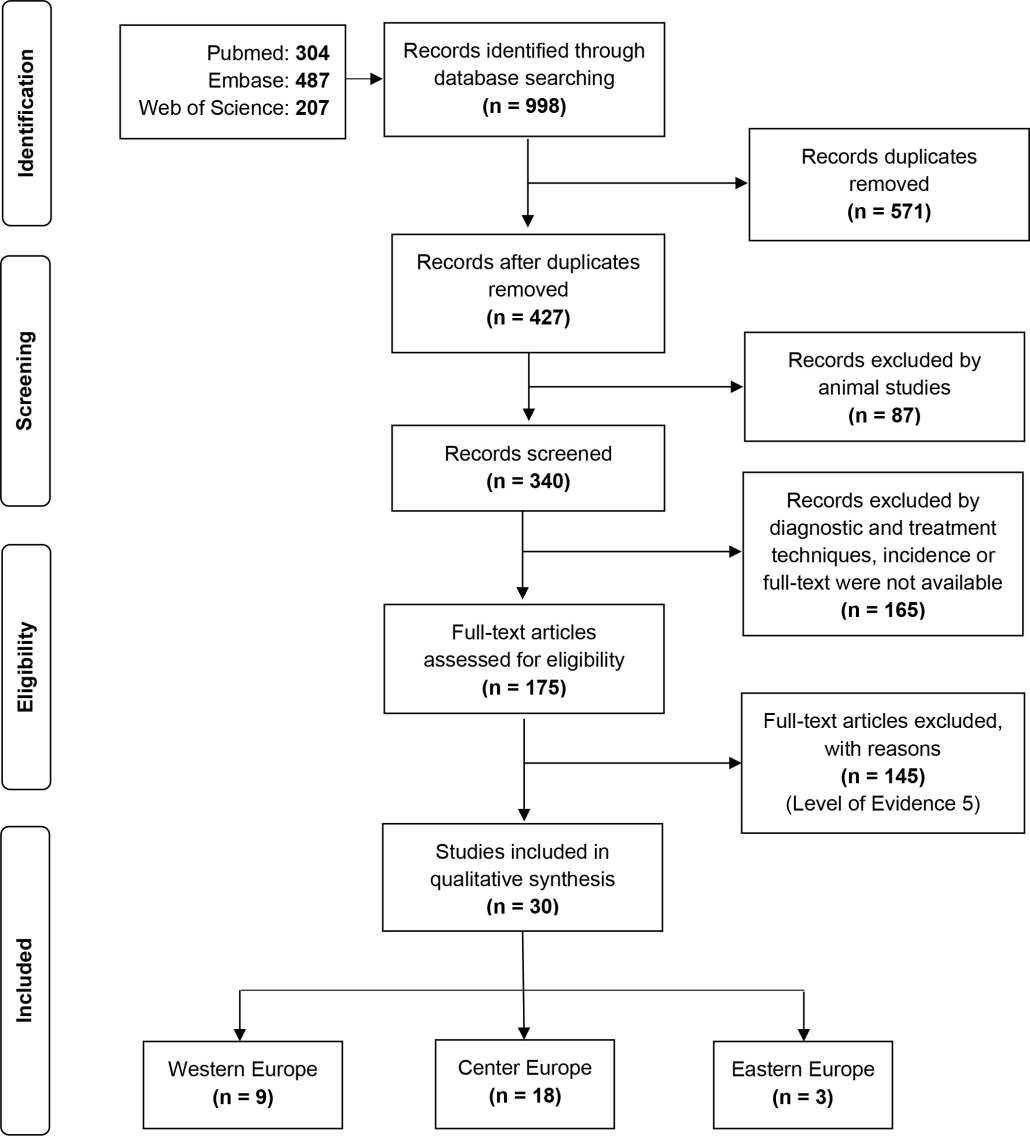
PRISMA flowchart of identified and selected studies in the systematic review.

The initial search identified a total of 998 references that met the inclusion criteria: 304 on PubMed, 487 on Embase and 207 on the Web of Science. We first removed 571 duplicated records and 87 animal studies. Then, 165 articles about diagnostic and treatment techniques, incidence data or whose full-text papers were not available were excluded; and, 175 full-text articles were assessed for eligibility. A total of 145 of these papers were excluded because they were mainly case reports (OCEBM Level of Evidence 5, Grade of Recommendation D). Finally, 30 studies met our inclusion criteria and were included in the qualitative synthesis. **Fig 1** shows the modified PRISMA flowchart with searching process.

The articles were classified according to their origin in three European regions. The main characteristics of these studies are shown in **Tables 1–3**: **Table 1,** western Europe, 9 articles [6,26–33]; **Table 2,** central Europe, 18 articles [4, 34–50]; and **Table 3,** eastern Europe, 3 articles [51–53]. Even though the geographical origin was diverse, most studies were performed in Turkey (central Europe) (13 articles) [4,34,37–39,41–42,44–45,47–50] and in Greece (western Europe) (7 articles) [6,26,29–33].

**Table 1:** Principal results of CCHF studies in western Europe.

Author	Publication year	Study years	Country	Objective (related to CCHF seroprevalence in humans)	N	Risk factors	Seroprevalence of IgG: Value (%)
Antoniadis *et al*. [26]	1982	1980–1981	Greece (rural area, Northern Greece)	To determine the prevalence of CCHFV antibodies in a rural population of Northern Greece	65	Farming Living in Northern Greece (CCHF isolated in this zone from Rhipicephalus bursa since 1978)	4 (6.2)
Filipe *et al*. [27]	1985	1980	Portugal	To establish the seroprevalence of CCHFV virus in Southern Portugal	190	Living in certain areas of Southern Portugal	2 (1.1)
Palomar *et al*. [28]	2017	2010–2014	Spain	To evaluate the presence of antibodies against the virus in individuals exposed to tick bites	228	No risk factors found	0 (0.0)
Papa *et al*. [29]	2014	2012	Greece	To make a small-scale serologic survey in humans and animals in the area where CCHFV-positive tick had been detected	100	Ageing	6 (6.0)
Papa *et al*. [30]	2013	2010–2012	Greece (Western, border to Albania and Ionian Sea Coast)	To check in more detail the CCHFV situation in Thesprotia prefecture (western region, border with Albania) and find out any risk factors associated with seropositivity	166	Ruminants husbandry Slaughtering Ageing	24 (14.4)
Papa *et al*. [31]	2011	2008–2009	Greece (Eastern, border to Bulgaria)	To determine the prevalence of CCHFV antibodies in the human population of Northeastern Greece	1178	Female sex Ageing Ruminants husbandry Slaughtering Tick exposure	37 (3.1)
Sargianou *et al*. [32]	2013	2012	Greece (Coast of the Gulf of Corinth)	To estimate the seroprevalence of CCHFV in humans in Achaia Prefecture, Greece, and to assess risk factors in seropositivity	207	Agropastoral occupation Ruminants (especially with sheep) Living at an altitude of ≥400m	7 (3.4)
Sidira *et al*. [33]	2013	2010–2011	Greece (Northern coast of the Aegean Sea)	To estimate the CCHFV seroprevalence among humans residing in the prefecture of Imathia, and the neighbouring prefecture of Pella, and to investigate demographics and probable risk factors associated with the seropositivity	277	Tick exposure Residence in a hilly territory Ageing Agropastoral occupation	6 (2.2)
Sidira *et al*. [6]	2012	2009–2010	Greece	To estimate endemic areas CCHF in Greece	1611	Slaughtering Agropastoral occupation Ruminants husbandry	68 (4.2)

**Table 2:** Main data of CCHF in studies in center Europe.

Author	Publication year	Study years	Country	Objective (related to CCHF seroprevalence in humans)	N	Risk factors	Seroprevalence of IgG: Value (%)
Bayram *et al*. [34]	2017	2012	Turkey (Eastern region, border with Iran)	To determine the seroprevalence of CCHFV in individuals with a high risk of acquiring CCHF disease in Van province	368	No risk factors found	53 (14.4)
Bodur *et al*. [4]	2012	2009–2010	Turkey	To investigate the seroprevalence of CCHFV infection in a sufficiently large sample representative of the region affected during the outbreak of 2011 in Turkey	3557	Ageing Low level of education Agropastoral occupation Tick exposure	356 (10.0)
Christova *et al*. [35]	2017	2015	Bulgaria	To estimate the prevalence of IgG antibodies to CCHFV and hantaviruses, as stable and long persisting antibodies, in general human population of Bulgaria	1500	Living in southeastern Bulgaria (especially in the Haskovo district) Ageing Ruminant husbandry Tick exposure	55 (3.7)
Christova *et al*. [36]	2013	2011	Bulgaria	To estimate the situation on CCHFV seroprevalence in both disease-endemic and -nonendemic areas in Bulgaria	1018	Tick exposure Ageing Living in the Black Sea Coast (Burgas District)	28 (2.8)
Cikman *et al*. [37]	2016	Not specified	Turkey (North-Eastern region)	To determine the seroprevalence and risk factors associated with Crimean-Congo hemorrhagic fever virus (CCHFV) in residents of North-Eastern, Turkey	372	Ruminant husbandry Living in rural areas Tick exposure	59 (15.8)
Ergönül *et al*. [38]	2006	2003	Turkey	To determine the seroprevalence of CCHFV, among veterinarians in a highly endemic and a non-endemic region for these infections in Turkey	83	Percutaneous injuries in veterinarians	1 (1.2)
Ertugrul *et al*. [39]	2012	Not specified	Turkey (Western region, Aegean Sea Coast)	To determine the rate of specific IgG seropositivity against the virus and the contributory factors	429	Age <34 years Tick exposure Ruminant husbandry Female sex	84 (19.6)
Fajs *et al*. [40]	2014	2012	Kosovo (Serbia for the WHO)	To determine the prevalence of CCHF in Kosovo	1105	Living in the Southwestern Serbia (hyperendemic regions) Ageing Male sex	44 (4.0)
Gargili *et al*. [41]	2011	2008–2009	Turkey (Western Turkey, border with Bulgaria)	To estimate whether there is an immune-protection in the region as a result of a stablished infection and not a recent spread of infected ticks into the area	193	Male sex Ageing Living in the Black Sea Coast	21 (10.9)
Gazi *et al*. [42]	2016	2011–2013	Turkey (rural part of Western Turkey)	To determine the seroprevalence of CCHFV among the rural residents of Manisa region, Turkey, and to identify the associated risk factors	324	Ageing	12 (3.7)
Gergova *et al*. [43]	2014	2011–2012	Bulgaria (Southeastern region)	To determine the seroprevalence of CCHFV in endemic areas of Bulgaria	751	Tick exposure Ruminant husbandry	24 (3.2)
Gozel *et al*. [44]	2013	2012	Turkey (Research Hospital, Centre-North Turkey)	To analyze the serum seropositivity for CCHFV IgM and IgG of all healthcare workers at risk, and to determine the possible risk factors	190	Visiting an endemic region	1 (0.5)
Gunes *et al*. [45]	2009	2006	Turkey (Centre-North region)	To determine the seroprevalence of CCHFV in a high-risk population	782	Ageing Tick exposure Contact with livestock	100 (12.8)
Horváth *et al*. [46]	1976	1972–1975	Hungary	To determine the seroprevalence of CCHFV in Hungary	587	Living in the Eastern Hungary, border to Rumania (Hajdú-Bihar county)	17 (2.8)
Koksal *et al*. [47]	2014	2004–2008	Turkey (Eastern Black Sea regions)	To determine the seroprevalence of CCHF infection and risk factors for disease in people living in the same environment with confirmed patients, either as household members or in the immediate neighbourhood, in the endemic area in the Black Sea region of Turkey	625	Ageing Ruminant husbandry Tick exposure Living rural areas	85 (13.6)
Tekin *et al*. [48]	2010	Not specified	Turkey (Northern region)	To determine the seroprevalence of CCHFV in humans in the province of Tokat (Centre-Northern region)	715	Contact with animals (not specified) Relatives of patients with CCHF (Airborne transmission?)	69 (9.6)
Temocin *et al*. [49]	2018	2016	Turkey (Centre region)	To determine the seroprevalence of CCHF disease among healthcare workers in a hospital in an endemic region, and to present the risk factors for healthcare workers	112	Percutaneous injuries in HCW	2 (1.8)
Yagci-Caglayik *et al*. [50]	2013	Not specified	Turkey	To estimate the CCHFV seroprevalence in apparently healthy adult population living in urban and rural areas of seven geographically representative provinces of Turkey and to find out the risk factors associated with the seropositivity	1066	Ageing Low level of education Male sex Farming Living in a house of adobe	25 (2.3)

**Table 3:** Main data of CCHF in studies in eastern Europe.

Author	Publication year	Study years	Country	Objectives (related to CCHF seroprevalence in humans)	N	Risk factors	Seroprevalence of IgG: Value (%)
Abdiyeva *et al*. [51]	2019	2014–2015	Kazakhstan	To detect the seroprevalence of CCHFV in patients with fever of unknown origin in endemic and non-endemic oblasts of Kazakhstan	802	Agro-pastoral occupation Ruminant and other livestock (except pigs) husbandry Living in rural areas	102 (12.7)
Greiner *et al*. [52]	2016	2014	Georgia (12 rural affected communities)	To determine CCHF seroprevalence, identify risk factors, and document CCHF-related knowledge, attitudes, and practices	444	Agro-pastoral occupation Animal husbandry Tick exposure	12 (2.8)
Magnaval *et al*. [53]	2011	2007	Russian Federation (Northeastern Siberia)	To determine the seroprevalence of nine zoonoses in Viljujsk, a Northern city in the Republic of Sakha (Eastern Siberia)	90	No risk factors found	10 (11.1)

The oldest article was published by Horvath *et al*. in 1979 [46], and the most recent was published by Abdiyeva *et al*. in 2019 [51]. All articles were cross-sectional studies (descriptive studies). The study design that was generally used was to assess the prevalence of a disease in a population (prevalence study), which has an OCEBM Level of Evidence 4, Grade of Recommendation C.

### Seroprevalence

**Fig 2:**
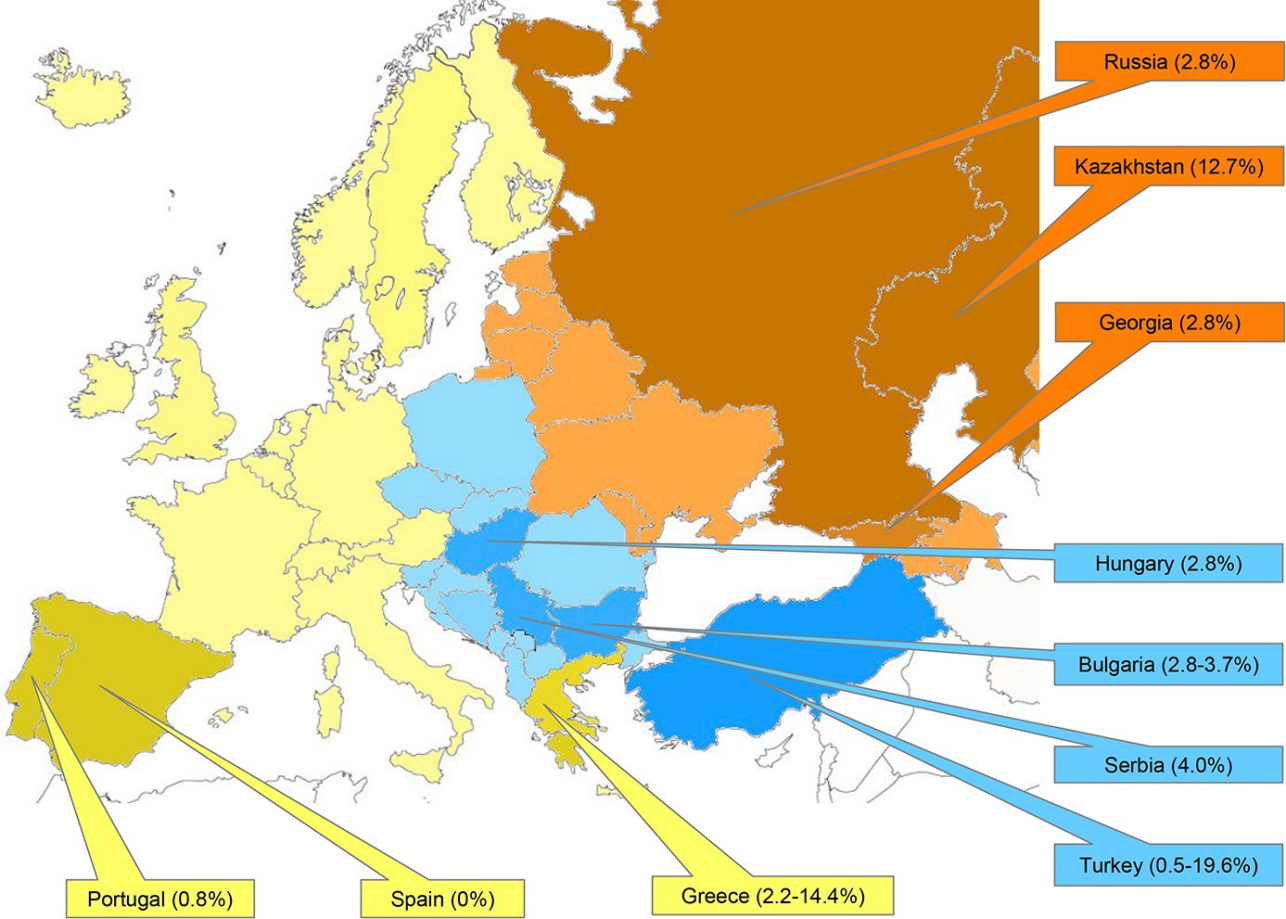
Seroprevalence of CCHFV in Western Europe (yellow), Center Europe (blue) and Eastern Europe (orange).

**Fig 2** shows in a Map the seroprevalence of CCHFV in the different European Regions.

**Table 4** summarizes the main quantitative data collected from the studies that were analyzed: the sample sizes and IgG antibody levels (seroprevalence).

**Table 4:** Estimated seroprevalence of CCHFV in the European Regions*.

**Western Europe**		
Greece	3604	2.2–14.4 ± 1
Portugal	258	0.8 ± 3
Spain	228	0.0
**Center Europe**		
Bulgaria	3269	2.8–3.7 ± 1
Hungary	587	2.8 ± 1
Serbia	1105	4.0 ± 1
Turkey	8816	0.5–19.6 ± 1
**Eastern Europe**		
Georgia	444	2.8 ± 2
Kazakhstan	802	12.7 ± 2
Russian Federation	90	11.1 ± 6

*95% Confidence Intervals (CI) for a proportion.

* References in Tables 1, 2 & **3**

The sample sizes varied from 3557 individuals in a study conducted in Turkey [4] to 65 individuals in a study conducted in Greece by Antoniadis *et al*. [26]. The reported seroprevalence was between 0% in Spain [28] and 19.6% in Turkey [39]. We analyzed each European region independently as follows.

#### Western Europe

The lowest seroprevalence was observed in Spain 0% [28] and Portugal 1.1% [27]. The seven included studies that were performed in different geographical areas of Greece obtained seroprevalence ranging between 2.2% [33] and 14.4% [30]. Nevertheless, six of the seven studies included a reported prevalence between 2.2% and 6.2% (2.2% [33], 3.1% [31], 3.4% [32], 4.2% [6], 6% [29], and 6.2% [26]).

#### Central Europe

The studies conducted in Turkey also showed high variability. Most studies showed a seroprevalence that ranged from low values (0.5% [44], 1.2% [38], 1.8% [49], 2.3% [50], and 3.7% [42]) to moderate values (9.6% [48], 10% [4], 10.9% [41], 12.8% [45], 13.6% [47], 14.4% [34], and 15.8% [37]. One study showed a much higher prevalence (19.6% [39]), but this study was performed in an endemic area in southwestern Turkey. Bulgarian studies reported a similar seroprevalence: 2.8% [36], 3,2% [43], and 3.7% [35]. Additionally, Hungary (2.8% [46]) and Kosovo (4% [40]) reported a similar seroprevalence.

#### Russia and western Asia

Georgia shows a prevalence similar to that in eastern European countries (2.8%,[52]), while the Russian Federation (11.1%,[53]) and Kazakhstan (12.7%,[51]) show a prevalence closer to that found in Turkish areas, with a moderate/high prevalence.

### Risk factors

Major risk factors, such as occupations associated with animal husbandry (especially of sheep and goats) [6,29,31,45,47–48,51–52] and agricultural and agropastoral activities [4,6,32,45,50,52] were identified in this study (no matter the geographical zone). Also another major risk factor was the tick exposure; tick exposure includes direct physical contact, tick bites, tick removal from people and animals and exposure to ticks around the working and home environments [12,31,33,35–37,39,43,45,47–48,52]. To a lesser extent, health care workers (physicians and nurses), veterinarians [38,44,48–49] and individuals with slaughtering-associated jobs [6,30,31] were also more likely to have the presence of CCHFV IgG antibodies.

Minor risk factors, such as gender and aging (risk markers) have been reported in some studies [4,29,31,33,35–36,39–42,45,47,50] in association with the presence of antibodies, but the heterogeneity of the studies and the populations that were evaluated prevent us from exhaustively affirming these results. Other risk factors that were evaluated were related to geographical aspects, such as residence in a hilly territory/living at an altitude [32–33], living in rural *vs* urban areas [26,37,47,50], in adobe houses [51], geographic regions as Black Sea Coast [36–41] and others [27,31,35,48] or endemic areas [40,43,47,49].

Some factors such as airborne transmission were also evaluated[48], but a causal association could not be determined, as the design of this study was not analytical.

## Discussion

In the recent years, the epidemiology of vector-borne diseases is changing due to diverse factors, especially linked to the global warming phenomena [54–55]. In Europe, a rise on the prevalence of most important tick-borne infections are mainly due to tick-borne encephalitis and Lyme borreliosis in Central and Eastern Europe [56,57], and the emergence of the CCHFV in Southwestern Europe [21].

In this systematic review, we have realized an exhaustive and comparative analysis about the human seroprevalence of CCHFV in the WHO European Region countries (http://www.euro.who.int/en/countries). We have established three different areas (Center, Western and Eastern Europe) in order to evaluate the progression of tick-borne infection in the continent and to estimate which geographical regions are particularly at risk. This topic has been the subject of other systematic reviews based in specific subgroups of patients, such as travelers or pregnant women [58–59]. Likewise, a recently published systematic review, realized by Nasirian H. [60] discusses some of the CCHF seroprevalence studies that we also present in this systematic review. Nasirian H. study [60] collects global CCHFV seroprevalence data (no IgG—IgM antibodies difference) in humans and animals from different areas of the world, while our study is limited to CCHF seroprevalence for IgG antibodies at the WHO European Regions.

The eastern region of Europe is well known as the first site where this infection was reported [2]. Currently, CCHF continues to be endemic in the Russian Federation and in other countries of the former Soviet Union, though the real prevalence of CCHF is difficult to estimate because of the low number of seroprevalence studies. Nevertheless, the studies included in the analysis found values above 10% in Kazakhstan [51] and in the north of Russia [53].

In the central European region, human seroprevalences above 5% were also seen. The northeast of Turkey, especially the areas surrounding the Black Sea (eastern Anatolia), are classically described as highly endemic (high prevalence and incidence rates) for this infection [37,47]. However, we found the highest seroprevalence (19.6%) in a study performed on the Aegean Sea coast, which is not considered a CCHFV endemic zone. Balkan countries are also considered endemic [35,40,43,61], but their seroprevalence is lower than that in Turkey. Other countries in central Europe, such as Hungary, are not considered endemic for this infection, though seropositivity for CCHFV antibodies has been described since 1976 [46]. Recent studies have shown that this zoonosis is also circulating in animals in countries such as Romania and the former Yugoslav Republic of Macedonia [62–64].

The Western region the WHO/European Region had the lowest seroprevalence values for this infection. The infection has been documented in this area since the 1980s, when Antoniadis *et al*. [26] and Filipe *et al*. [27] demonstrated seropositivity in healthy humans in Greece and Portugal, respectively. Nevertheless, it was not until 2010 that the first and only autochthonous case was reported in Greece [20]. Additionally in 2010, the epidemiological alert in southwestern Europe increased after evidence of virus circulation in ticks belonging to the *Hyalomma marginatum* species were retrieved from a wild red deer in western Spain [65], near the Portuguese border. Six years later, first human autochthonous case was reported in Spain, a 62-year-old man that, after traveling to a little village at Central-Western Spain, presented at the Emergency Department with a severe viral hemorrhagic fever and died on the ninth day of illness. Four days later, a secondary (non-fatal) case due to a nosocomial transmission was also reported [21,66]. Since then, other two cases have also been reported in the Western Spain [67], reflecting probably only the visible part of the iceberg. Even though a recent study found no seroprevalence in humans in Spain [28], larger studies of seroprevalence need to be carried out in humans to corroborate the existence of an undetected circulation occurring in other areas of Spain and in neighboring countries, like Portugal, France, Italy or Malta where the vector tick exists and the weather conditions are favorable for the dissemination of this vector-borne disease.

Major risk factors have been well documented to be associated to tick exposure [12,31,33,35–37,39,43,45,47–48,52] in endemic regions, especially in the individuals involved in ungulate husbandry [6,29,31,45,47–48,51–52] and/or agropastoral activities [4,6,32,45,50,52]. The outbreaks emerge mainly in the spring and summer (May to October) [1]. Nevertheless, global migratory human movements, bird migrations from Africa, weather changes, global warming [55], and the presence of the main vector, *Hyalomma marginatum* ticks, in most countries of Europe might result in a situation in which this infection appears in other periods, spreads to new areas, increases in incidence, and becomes a diagnosis to be ruled-out in patients with hemorrhagic fever, even when a tick bite history cannot be documented.

The main limitation of this study was the heterogenicity of the studies and the lack of published seroprevalence investigations of some regions with elevated endemicity, especially at Northeastern Europe (Russia, Ukraine, between others). For this reason, a meta-analysis could not be performed. However, an extensive research in the main databases was performed, only excluding studies with low level of evidence. Nevertheless, a qualitative review improves the current lack of information. More studies are necessary to obtain conclusive evidence. The risks of bias (methodological and clinical) may have affected the results of our qualitative review. Despite these limitations, this systematic review sought to analyze the available information to date related to the epidemiology of CCHF in WHO/ European Region.

This systematic review suggests the following conclusions. i) The highest values of CCHFV seroprevalence are found in Turkey, the Russian Federation, and Kazakhstan. ii) Greece has a high seroprevalence, though only one death associated with CCHFV has been reported [20]. This fact contrasts with the neighboring countries, such as Balkan countries and Turkey, where the rate of severe infections seems to be higher. iii) Extensive studies should be developed in European countries to establish the actual epidemiological situation and to take additional preventive measures for the future.

## Supporting information

S1 Checklist PRISMA checklist. (DOC)
